# Green Synthesis, Characterization and Application of Natural Product Coated Magnetite Nanoparticles for Wastewater Treatment

**DOI:** 10.3390/nano10081615

**Published:** 2020-08-18

**Authors:** Chanchal Das, Subhadeep Sen, Tejinder Singh, Tanmoy Ghosh, Subha Sankar Paul, Tae Wan Kim, Seob Jeon, Dilip K. Maiti, Jungkyun Im, Goutam Biswas

**Affiliations:** 1 Department of Chemistry, Cooch Behar Panchanan Barma University, Vivekananda Street, Cooch Behar 736101, West Bengal, India; chanchaldas453@gmail.com (C.D.); sensubhadeep07@gmail.com (S.S.); 2 Department of Electronic Materials and Devices Engineering, Soonchunhyang University, Asan 31538, Korea; tejinder@sch.ac.kr; 3 Department of Chemistry, University of Calcutta, 92 A. P. C. Road, Kolkata 700009, India; tanmoyghoshchem@gmail.com; 4 Singapore Center for Environmental Life Sciences Engineering, Nanyang Technological University, Singapore 637551, Singapore; sankarsuvo@gmail.com; 5 Department of Medical Life Science, Soonchunhyang University, Asan 31538, Korea; ktwdreem@naver.com; 6 Department of Obstetrics and Gynecology, College of Medicine, Soonchunhyang University, Cheonan Hospital, Cheonan 31151, Korea; sjeon4595@gmail.com; 7 Department of Chemical Engineering, Soonchunhyang University, Asan 31538, Korea

**Keywords:** magnetite nanoparticles, wastewater treatment, antibacterial, toxic metal removal, dye adsorption

## Abstract

Adsorption of organic pollutants, toxic metal ions, and removal of harmful bacteria can give us clean and pure drinkable water from wastewater resources. Respective magnetite nanoparticles (MNPs) were synthesized using a cheaper and greener way in an open-air environment with the use of crude latex of *Jatropha curcas* (JC) and leaf extract of *Cinnamomum tamala* (CT). Characterization of MNPs had been performed by dynamic light scattering (DLS), Ultraviolet-visible (UV-vis) spectroscopy, Fourier-transform infrared (FTIR) spectroscopy, powdered X-ray diffraction (XRD), and field emission scanning electron microscope (FE-SEM). The size ranges of the synthesized MNPs were observed in between 20–42 nm for JC-Fe_3_O_4_ and within 26–35 nm for CT-Fe_3_O_4_ by FE-SEM images. The effect of synthesized magnetic nanoparticles in wastewater treatment (bacterial portion), dye adsorption, toxic metal removal as well as antibacterial, antioxidant, and cytotoxic activities were studied. This purification will lead to an increase in the resources of pure drinking water in the future.

## 1. Introduction

The freshwater scarcity and water pollution problems have been increasingly growing worldwide in the last several years [1]. At present, around 3.1% of deaths happening every year, which is over 1.7 million all over the world, are caused just because of unsafe and lack of reliable sources of drinkable water [2]. It is estimated that more than 57% of the world’s population will have difficulties in accessing water throughout the year by 2050 [1]. Water pollution is the principal cause for the lack of suitable drinking water resources. In every growing nation, importance on industry and agricultural evolution leads to contamination of water with harmful organic pollutants and metals like cobalt (Co), copper (Cu), nickel (Ni), lead (Pb), zinc (Zn), arsenic (As), cadmium (Cd), chromium (Cr), and mercury (Hg) [3,4]. Another important problem is waterborne harmful bacteria viz. *Salmonella typhosa* (typhoid), *Vibrio cholerae* (cholera), *Escherichia coli* (diarrhea), etc. This necessitates the urgency to remove toxins and impurities from aquatic environments rapidly and efficiently, by means of technological advancement. There are many physical, chemical, and biological purification methods available to remove organic pollutants, heavy metals, and harmful bacteria from wastewater. The adsorption technique is the most widely used physical technique, efficient to remove inorganic and organic pollutants from wastewater. Many natural and synthetic adsorbents have already been developed [5,6,7]. Among them, activated carbon, rice husk, carbon nanotubes, mesoporous silica, curcubiturils etc. are well-established materials [8,9,10].

In the last few decades, nanoparticles have emerged as one of the versatile tools for catalysis [11], biosensors [12], cell labelling [13], medicines [14], solar cells [15], fuel cells [16], photonic band gap materials [17], and in many more applications.

Nanoparticles (NPs) have very large surface area. Hence, they are more efficient to bind with various molecules [18]. The advantage of magnetite nanoparticles (MNPs) lies in its super paramagnetic behavior. Hence, MNPs have the added control of changing the concentration of nanoparticles by the simple use of the magnet. MNPs have been recently reported to be developed for the purification of wastewater [19,20,21]. The use of the magnetic field after purification to remove the nanoparticles makes the purification process simpler, cost-efficient, and safe to handle. MNPs are synthesized easily by different methods, among which the most popular and common is by the co-precipitation method [22,23]. The main drawback of MNPs is the aggregation, which is formed due to the lack of stabilizing agents. By suitable surface modifications, they can be produced in variable nanosize range and, therefore, can be useful in many applications like theranostic treatment and phototherapeutic treatment for heavy metal removal from groundwater [22,24]. Various surface modifier agents are used like polyethylene glycol (PEG) [25], SiO_2_ [26], ionic liquids [27], and extracts of different parts of plants to modify the size, stability from aggregation, and the biocompatibility of the MNPs [28,29]. MNPs can be conveniently prepared by the greener way and at low cost [30,31,32]. The multimodal properties of the MNPs have been utilized in many applications [33].

The Indian spice *Cinnamomum tamala* leaf is known for its antioxidant [34], as well as antibacterial properties [35], and similar properties are found in the case of *Jatropha curcas* latex as well [36,37]. Several MNPs have been synthesized with natural product extracts like neem [38], bhringraj [39], curcumin [40], etc. and are utilized as capping agents as well as stabilizing agents for nanoparticles. In all the cases, the antibacterial property, antioxidant property, and anti-cancer activity are some of the commonly explored properties.

Herein, we report the synthesis of two different nanoparticles CT-Fe_3_O_4_ and JC-Fe_3_O_4_ coated with *Cinnamomum tamala* (CT) leaves and *Jatropha curcas* (JC) latex extract respectively. To date, no literature has reported the green syntheses of above two natural products-based MNPs. The two nanoparticles CT-Fe_3_O_4_ and JC-Fe_3_O_4_ are further characterized, and their applications in wastewater treatment as well as their antibacterial activity, antioxidant activity, and cytotoxicity have been explored.

## 2. Chemicals and Experimental Methods

### 2.1. Materials Used

*Cinnamomum tamala* leaves were procured commercially, whereas fresh *Jatropha curcas* latex was collected from Alipurduar district, West Bengal, India. Commercially available iron (II) chloride (FeCl_2_·H_2_O), iron (III) chloride (anh. FeCl_3_) and the other reagents were of analytical grade and utilized without further purification. All the solutions were made by freshly prepared deionised water (DW). High glucose Dulbecco’s modified Eagle’s medium (DMEM), Roswell Park Memorial Institute (RPMI) 1640, Dulbecco’s phosphate-buffered saline (DPBS, pH 7.4), fetal bovine serum (FBS), and trypsin/EDTA were procured from Gibco™, Thermo Fischer Scientific (Loughborough, UK). 3-(4,5-dimethylthiazol-2-yl)-2,5-diphenyl tetrazolium bromide (MTT) was purchased from Sigma Aldrich, Goyang, Korea. MilliQ purified water (18.2 MΩ) was used to prepare all biological solutions.

Cancer cells, both SW480 and HeLa, were incubated at 37 °C in a humidified 5% CO_2_ environment in RPMI 1640 and 10% (*v*/*v*) FBS with penicillin.

### 2.2. Preparation of Aqueous Extract of CT Leaf and 3% (v/v) JC Latex Extract

Commercially available dried 20 g CT leaves were crushed into small pieces and washed with DW to remove any impurities. Then around 70 mL DW was added and boiled for 20 min, filtered through Whatman-40 filter paper, and straw yellow leaf-extract was found to be around 30 mL.

Freshly collected 3 mL of JC crude latex was mixed with 97 mL of DW to get 3% (*v*/*v*) solution of it. The optimum concentration of 3% latex solution was prepared because greater concentration makes it insoluble in water, and lower concentration was insufficient for nanoparticles synthesis [37].

### 2.3. Preparation of CT-Fe_3_O_4_ NPs

2.03 g FeCl_2_·H_2_O and 5.19 g FeCl_3_ (anh.) were mixed, and 160 mL deionised water was added, stirred for 30 min, followed by the addition of optimum 40 mL CT aqueous leaf extract. Then 5.12 g NaOH in 80 mL DW was added to the mixture (to complete the precipitation) and the black solution was stirred further for about 60 min to complete the reaction and get the final black precipitate. Centrifugation was performed followed by washing with deionised water, repeatedly for 2–3 times to get the nanoparticles and was dried in vacuum desiccator for overnight to get the nanoparticles.

### 2.4. Preparation of JC-Fe_3_O_4_ NPs

One-hundred milliters of DW was added to the mixture of 1.27 g FeCl_2_·H_2_O and 3.24 g FeCl_3_ (anh.), stirred for 30 min, followed by the addition of 125 mL 3% JC latex; 3.2 g NaOH in 50 mL DW was added to the mixture and stirred further for 50 min to complete the reaction. Centrifugate was washed with DW repeatedly two to three times, and then dried in a vacuum desiccator overnight to get the final nanopowder.

### 2.5. Characterization of CT-Fe_3_O_4_ and JC-Fe_3_O_4_ NPs

DLS (model ZETASIZER Nano Series Nano ZS, Malvern Panalytical, Malvern, UK) technique was used to get a rough idea about the average size and particle size distribution of respective nanoparticles (hydrated sphere). UV-Vis spectroscopy had been done by using Perkin Elmar instrument, Waltham, MA, USA). Clustered metallic oxides were investigated by powdered X-ray diffractometry using Rigaku Miniflex 600 (Japan) equipped with copper X-Ray tube (Cu-Kα1,2 radiation) and NaI (Tl) scintillating detector. The surface morphology of the MNPs was studied using (FE-SEM, MIRA II LMH, Tescan, USA). Fourier-transform infrared spectroscopy (FTIR) was performed by using IR spectrometer (JASCO FT/IR-4600, Tokyo, Japan) with a resolution of 4 cm^−1^ and the scan range of 650–4000 cm^−1^. Magnetization measurements were performed on SQUID magnetometer (Quantum Design MPMS-7) from KBSI (Daejeon, South Korea).

### 2.6. Dye Adsorption Experiment

The dye adsorption study of the CT-Fe_3_O_4_ and JC-Fe_3_O_4_ NPs was performed by taking a known concentration of methylene blue (MB) (200 mg/L) solution and a definite quantity of MNPs (50 mg) in a conical flask (at pH~7), shaken thoroughly for 120 min at room temperature. At an interval of 20 min, the optical density (OD) was checked for each solution at 660 nm for adsorption kinetic study. For another set of experiments, a series of different initial concentrations (200 mg/L–500 mg/L) of MB were shaken (at pH~7, room temperature) with MNPs (50 mg) for 120 min, and the optical density (OD) was checked for each solution for adsorption isotherm study. While collecting the samples, the nanoparticles were removed by application of an external magnetic field [41].

Removal of MB dye (%) = [(*C_o_* − *C_e_*)/*C_o_*] × 100 (1)

*C_o_* = initial concentration of MB dye only, *C_e_* = concentration of MNPs solution (MB dye + MNPs) at equilibrium.

### 2.7. Toxic Metal Adsorption Experiment (with Concentration)

For metal adsorption study, we chose copper (II) acetate and cobalt (II) chloride as a source for copper (Cu^2+^) and cobalt (Co^2+^) ions, respectively. Five different concentrations (400 mg/L, 600 mg/L, 800 mg/L, 1200 mg/L, and 1400 mg/L) of both salt solutions were prepared (pH~7, room temperature). Two milligrams of CT-Fe_3_O_4_ NPs and 2.0 mg of JC-Fe_3_O_4_ NPs were added separately to each test tube containing 5 mL of each salt solution and were ultrasonicated for 120 min. Next the optical density (at 650 nm and 510 nm for Cu^2+^ and Co^2+^ solutions respectively) was checked for the solutions [42,43].

### 2.8. Antibacterial Assay for Wastewater Treatment

The sample pond water was collected from Cooch Behar district, West Bengal, India. Three conical flasks, each containing 10 mL of sample pond water, were treated separately with 0.05g CT-Fe_3_O_4_ and 0.05 g JC-Fe_3_O_4_ NPs alongside a blank experiment, and were shaken for 10 min. Then from the stirred solutions, 100 µL of the mixture was spread on Luria Broth (LB)-agar plates and were incubated for 24 h at 37 °C [44]. For the colony forming units (cfu) calculation, the same process was repeated, using different amounts (0.04 g, 0.02 g and 0.01 g) for each of CT-Fe_3_O_4_ and JC-Fe_3_O_4_ NPs.

### 2.9. Isolation and Characterization of Bacteria from the Collected Sample

The collected pond water was serially diluted from 10^−1^ to 10^−15^ times and each dilution was spread on the LB-agar plate using spread plate technique and incubated for 24 h at 37 °C. Next, one bacterial single colony was isolated and subcultured to obtain a pure culture. The pure culture was stored in 3 mL microcentrifuge tube for further assays (Disk Diffusion Test and Minimum inhibitory concentration (MIC) determination). Gram staining was performed on the isolated pure culture. Briefly, loopful of mother culture was taken, and smeared on the glass slide. The smear was air dried and fixed by passing over the flame. A few drops of crystal violet stain were added to the fixed smear and were kept for 1minute. Then a few drops of iodine solution were added. Next, alcohol washing was done for 25–30 s. Furthermore, a few drops of saffranine were added and kept for 1 min. After every step, excess stain was washed off with water and dried in air, and the slides were observed under a microscope.

#### 2.9.1. Disk Diffusion Test

One-hundred microlitres of bacterial suspension (0.5 McFarland Standard) was spread over the surface of the LB-Agar plate and allowed to dry for 10 min. Then two sterile paper disk (5 mm) saturated with 30 µL of CT-Fe_3_O_4_ and another with JC-Fe_3_O_4_ NPs (200 ppm) were placed in the culture medium. The plates were sealed by parafilm and incubated at 37 °C for 24 h, and the diameter of the resulting inhibition zone in every plate was measured [45,46]. All the experiments were performed in duplicates, and the results were expressed as mean values.

#### 2.9.2. Minimum Inhibitory Concentration (MIC) Determination

MIC of CT-Fe_3_O_4_ and JC-Fe_3_O_4_ NPs were determined by the broth dilution method using microliter-sized wells. The positive control sample contained 100 µL of bacteria inoculums with a culture medium in the absence of MNPs. The negative control contained 100 µL of culture medium in the absence of MNPs for monitoring sterility. Fifty microlitres of serially diluted CT-Fe_3_O_4_ and JC-Fe_3_O_4_ NPs were added to 10 columns containing 50 µL of culture medium (LB medium) to maintain the concentration sequence from 4000 ppm to 7.8 ppm. The standardized bacterial culture (1 × 10^5^ cfu/mL) was added in each well from columns 1–10. Further, the microlitre-sized wells were incubated at 37 °C for 24 h, and the resulting turbidity was observed. The MIC was determined where there was no visible growth of bacteria detected. For further confirmation, the turbidity was measured by optical density readings at 600 nm with a UV-Vis spectrophotometer [47,48].

#### 2.9.3. Antibacterial Assay

##### Bacteria Culture Preparation

The commercial bacterial stain of *E. coli* (ATCC 25922) and *S. aureus* (ATCC 29213) were grown in LB medium for 24 h at 37 °C, and optical density readings were compared to a 0.5 McFarland standard. Both the disk diffusion test and MIC were performed in the same procedure discussed before.

### 2.10. 2,2-Diphenyl-1-picrylhydrazyl (DPPH) Radical Scavenging Assay

Free radical scavenging activity was estimated by the DPPH scavenging assay [49,50]. 10.14 µM solution of DPPH in methanol was added to 500 µL MNPs solutions in methanol (total volume was 5 mL) in different concentrations (0.06 mg/mL, 0.25 mg/mL, 0.57 mg/mL, 1.00 mg/mL, 1.57 mg/mL) and the activity was observed at 517 nm after keeping the solutions in dark for about 30 min. The control sample was prepared without MNPs. Gallic acid was used as a positive control in all cases. The scavenging activity estimation was performed using the formula.

Scavenging activity (%) = [(*A_o_* − *A_s_*)/*A_o_*] × 100 (2)

where *A_o_* is the absorbance of the control (DPPH + methanol), and *A_s_* is the absorbance of the respective sample solutions (sample in methanol + DPPH solution).

### 2.11. Measurement of Cytotoxicity Using MTT Assay

SW480 and HeLa cells (4.5 × 10^3^ cells per well) were seeded into 96-well plates. After 24 h, the media was changed to non-serum RPMI 1640, and after a further 24 h, the cells were treated withJC-Fe_3_O_4_ and CT-Fe_3_O_4_ NPs at different concentrations. The treated cells were incubated for 48 h, washed with cold PBS, and then exposed to MTT with media for 4 h. The media was changed to DMSO, and the dissolved formazan dye was quantified by measuring the absorbance at 540 nm. As a control, untreated cells were examined in the same manner.

## 3. Results and Discussion

### 3.1. Synthesis of Nanoparticles

There are different procedures reported for the synthesis of MNPs by the co-precipitation methods, and most of them require an inert environment and, in some cases, an elevated temperature. Herein our study, we have reported the synthesis of JC-Fe_3_O_4_ and CT-Fe_3_O_4_ NPs at room temperature without inert gas environment and elevated temperature. In addition, we have replaced the use of ammonium hydroxide solution with NaOH solution and this collective approach makes our synthetic procedure much greener than the other reported methods because of environmentally friendly reagents, low toxicity, and biodegradable products [51]. This collective approach makes our synthetic procedure much greener than the methods reported hitherto in the literature.

### 3.2. Dynamic Light Scattering Experiment

**Figure 1:**
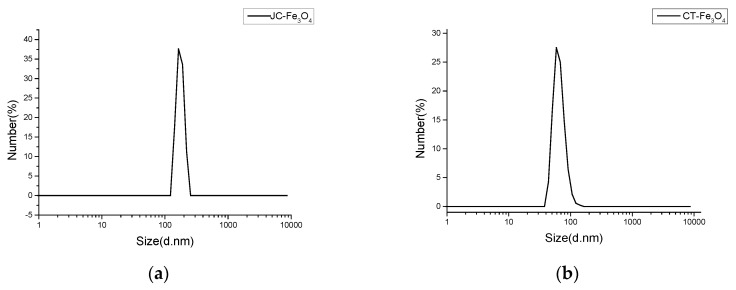
DLS curve of (**a**) JC-Fe_3_O_4_ nanoparticle; (**b**) CT-Fe_3_O_4_ nanoparticle.

Hydrodynamic size measurements are usually greater than the actual size measurements of the MNPs, which is due to the presence of extra hydrated layers attached on the surface. DLS experiment showed that hydrated MNPs had average sizes of around 154.2 nm for JC-Fe_3_O_4_ (Figure 1a) and around 65 nm for CT-Fe_3_O_4_ (Figure 1b). Hence, from the DLS graphs, we conclude that the formation of nanoparticles was completed in both the cases since the distributions are more even with a narrow distribution range.

### 3.3. UV-Visible Spectroscopy

**Figure 2:**
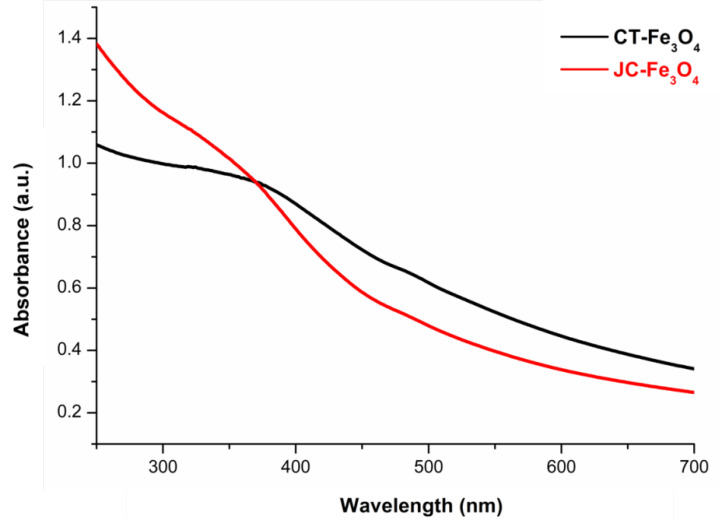
UV-Vis absorption spectra of CT-Fe_3_O_4_ and JC-Fe_3_O_4_ NPs.

The UV-Vis spectra (Figure 2) exhibited the characteristic continuous peak absorption of both the magnetite nanoparticles in the visible range; the absorption range was between 300–800 nm [52]. This confirms the formation of iron oxide nanoparticles. From the appearance of a broadband and the absence of any hump spectra, it may be concluded that not much size difference was present in the synthesized nanoparticles.

### 3.4. FTIR Spectroscopy

#### 3.4.1. FTIR Analysis of JC-Fe_3_O_4_ Nanoparticles

**Figure 3:**
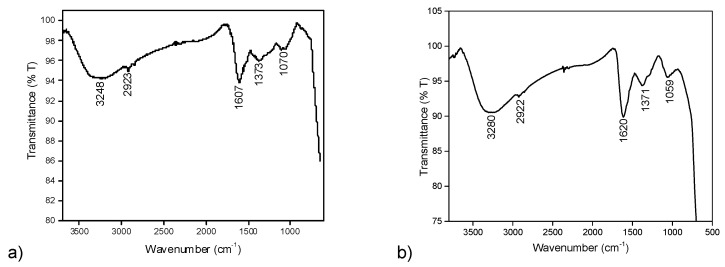
FTIR spectra of (**a**) JC-Fe_3_O_4_ and (**b**) CT-Fe_3_O_4_ NPs.

Next the FTIR analysis was performed to prove the presence of JC latex as the capping material for the synthesized MNPs. Synthesized JC-Fe_3_O_4_ NPs showed a strong absorption band at 1607 cm^−1^ (stretching vibration of C–N group), which was attributed to the binding of JC latex as the capping agent since this peak was also observed to be a significant peak in case of dried JC latex powder at 1618 cm^−1^ [37]. Other significant FTIR peaks showed the bands at 3248 cm^−1^ (N–H stretching for amides), 2923 cm^−1^ (secondary amine), 1373 cm^−1^ (–CO–stretching), and 1070 cm^−1^ (O–H stretching), which clearly proved the presence of protein/peptide on the nanoparticle binding surface. This data also fitted well with the previously reported *Jatropha curcas* extract capped nanoparticles [53] (Figure 3a).

#### 3.4.2. FTIR Analysis of CT-Fe_3_O_4_ Nanoparticles

Similarly, the FTIR peak analysis of CT-Fe_3_O_4_ NPs revealed that a broadband 3280 cm^−1^ was due to O–H stretching from the eugenol–OH present in the aqueous extract of CT leaf. The other significant bands at 1620 cm^−1^ (for carbonyl stretching) match well with the reported CT extract IR at 1638 cm^−1^ [54]; 2922 cm^−1^ (for C–H stretching) and 1059 cm^−1^ (for C–O stretching vibration) confirmed the formation of CT leaves extract-coated MNPs [55] (Figure 3b).

### 3.5. Powder XRD Analysis of JC-Fe_3_O_4_ and CT-Fe_3_O_4_ Nanoparticles

**Figure 4:**
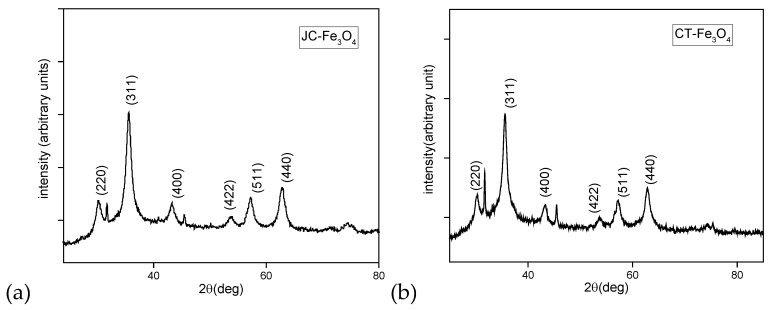
X-ray diffractometer patterns of MNPs (**a**) JC-Fe3O4 and (**b**) CT-Fe3O4.

The X-ray powder diffractograms of the JC-Fe_3_O_4_ (Figure 4a) showed a series of diffraction peaks at 2θ = 30°, 35°, 43°, 54°, 57°, and 63° and were assigned to (2 2 0), (3 1 1), (4 0 0), (4 2 2), (5 1 1), and (4 4 0) planes of cubic structures, which were in good accordance with the inverse cubic spinel phase of Fe_3_O_4_ (magnetite, JCPDS card no. 85-1436). Similarly, for CT-Fe_3_O_4_ (Figure 4b), the X-ray powder diffractograms showed a series of diffraction peaks at 2θ = 30°, 35°, 44°, 54°, 57°, and 63° and were assigned to (2 2 0), (3 1 1), (4 0 0), (4 2 2), (5 1 1), and (4 4 0) planes of cubic structures, which confirmed the same inverse cubic spinel phase of Fe_3_O_4_ NPs. These results were similar to those reported in the literature [56].

### 3.6. FE-SEM Analysis

**Figure 5:**
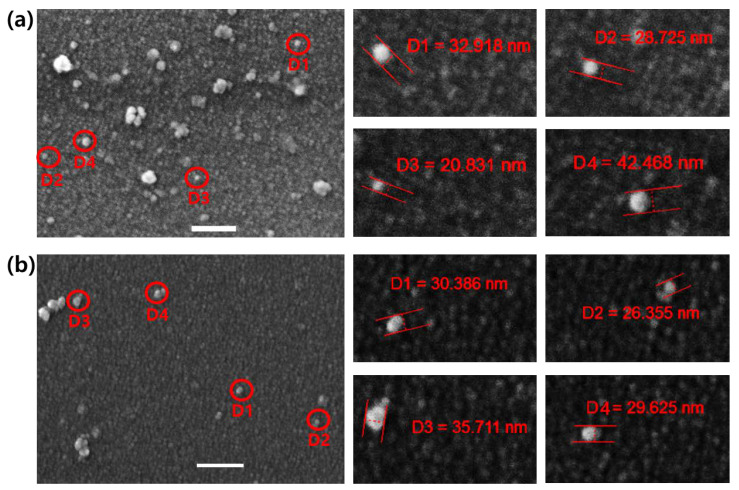
FE-SEM images showing the morphology of (**a**) JC-Fe_3_O_4_ and (**b**) CT-Fe_3_O_4_ NPs; Left: Single nanoparticles are marked with red circles. Right: Magnification of each single nanoparticle with the measured diameter (scale bar: 200 nm).

Analysis of FE-SEM images for JC-Fe_3_O_4_ and CT-Fe_3_O_4_ NPs showed the surface morphology of respective NPs was round-shaped. The size ranges for JC-Fe_3_O_4_ and CT-Fe_3_O_4_ were 20–42 nm (Figure 5a) and 26–35 nm (Figure 5b), respectively, and both were well surrounded by the respective green coating. The images confirmed that the formation of natural product-based nanoparticles had a spherical shape.

### 3.7. Dye Adsorption Study

The adsorption of MB as a model pollutant was performed to evaluate the adsorption ability of synthesized JC-Fe_3_O_4_ and CT-Fe_3_O_4_ NPs. Two model equilibrium adsorption isotherms, viz., Langmuir adsorption isotherm and Freundlich adsorption isotherm, were applied. For better understanding of the adsorption process, it is important to investigate the relevant kinetics; two different common kinetic models, pseudo-first-order and pseudo-second-order models were studied.

#### 3.7.1. Dye Adsorption Isotherm of MB Dye

The Langmuir isotherm accounts for the monolayer surface coverage of the adsorbents while the Freundlich isotherm defines for multilayer adsorption. The linear form of Langmuir and Freundlich isotherm equations are depicted as:

Langmuir model: *C_e_*/*Q_e_* = 1/(*Q_m_K_L_*) + *C_e_*/*Q_m_* (3)

Freundlich model: log *Q_e_* = log *K_F_* + (1/*n*) log *C_e_* (4)

The adsorption capacity is calculated by the equation: *Q_e_* = (*C_o_*−*C_e_*) × *V*/*m* (5)

Another adsorption parameter, *R_L_*, correlation factor at equilibrium, is also calculated for adsorption of MB on for Langmuir isotherm using the equation:

*R_L_*= 1/(1+*C_o_K_L_*) (6)

For 0 < *R_L_* < 1, adsorption process is satisfactory, and for *R_L_* ≥ 1 it is unfavourable.

Where, *C_o_* = initial concentration of adsorbate in mg/L, *C_e_* = equilibrium concentration of (adsorbate + adsorbent), *Q_e_* = adsorption capacity in mg/g, *K_L_* = Langmuir constant, *Q_m_* = maximum adsorption capacity, *K_F_* = Freundlich constant, *n* = separation factor, *V* = total volume of the solution in L, *m* = amount of adsorbent in g.

**Figure 6:**
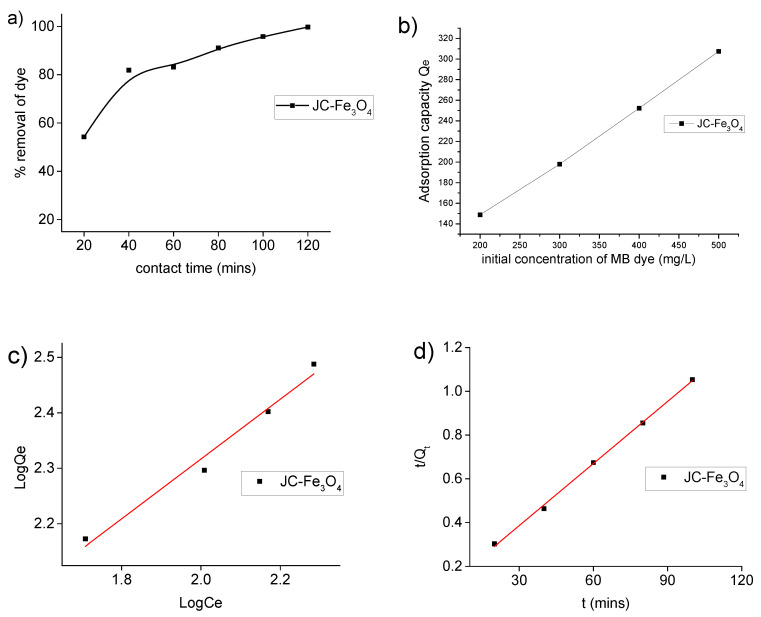
Plot of (**a**) % removal of MB dye with contact time; (**b**) adsorption capacity (*Qe*) of MB dye with concentration; (**c**) Freundlich adsorption isotherm model (Log *Q_e_* vs Log *C_e_*); (**d**) Pseudo-second-order kinetic model for adsorption *t*/*Q_t_* vs. *t* (mins) of JC-Fe_3_O_4_ NPs.

It was observed from Figure 6a that the removal percentage of MB dye (200 mg/L) increased with respect to contact time in the presence of JC-Fe_3_O_4_ NPs. The absorption peak of MB dye at 660 nm gradually decreased with time and after 120 min the color decreased considerably. Hence, taking 120 min as the optimum time, we performed further experiments. From Figure 6b, the adsorption capacity of the MNPs increase with increasing concentration of the dye.

From Figure 6b, it is evident that the dye removal capacity of JC-Fe_3_O_4_ is dependent on the initial concentration of the MB. Thus, the removal capacity is increasing with the increase in the initial concentration of dye. Analysis of data from the plot of [log *Qe* against log *Ce*] (Figure 6c) as tabulated in Table 1 supports that for MB dye adsorption, Freundlich model fits better than Langmuir model in case of JC-Fe_3_O_4_ NPs as the values of *R^2^* in the case of Freundlich model are much closer to 1.

#### 3.7.2. Adsorption Kinetics for MB Dye

Adsorption kinetics study for MB dye over synthesized JC-Fe_3_O_4_ and CT-Fe_3_O_4_ NPs wasinvestigated using pseudo-first-order and pseudo-second-order kinetic models. Pseudo-first order and pseudo-second order kinetics were investigated by the following equations [57]:

Pseudo-first-order: log (*Q_e_* − *Q_t_*) = log *Qe* − *K*_1_*t*/2.303 (7)

Pseudo-second-order: *t*/*Q_t_* = 1/*K*_2_*Q_e_*^2^ + t/*Q_e_* (8)

*Q_t_* was calculated by the equation: *Q_t_* = (*C_o_* − *C_t_*)*V*/*m* (9)

where, *K*_1_ = rate constant of first-order kinetics in min^−1^, *K*_2_ = rate constant of second order kinetics in g/mg·min, *Q_e_* = adsorption capacity at equilibrium in mg/g, and *Q_t_* = adsorption capacity in mg/g at time t.

All the experimental and calculated data based on the above kinetic models for adsorption of MB on JC-Fe_3_O_4_ NPs are presented in Table 2. From the results in Table 2 and Figure 6d, it is seen that although the correlation coefficient (*R*^2^) is almost similar for both the kinetic models, the experimental value of the adsorption capacity *Q_e_* (expt) agrees better with the calculated value *Qe* (cal) based on pseudo-second-order model than with pseudo-first-order model, indicating that our adsorption process follows the former model. No consistent results were observed for the dye adsorption on CT-Fe_3_O_4_ NPs.

### 3.8. Toxic Metal Adsorption Study (with Concentration)

**Figure 7:**
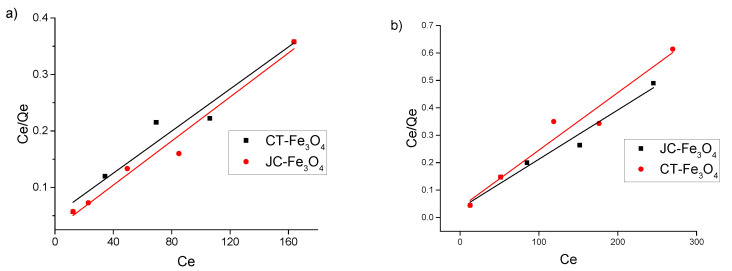
Langmuir isotherm plot for the removal of (**a**) Co^2+^ ions and (**b**) Cu^2+^ ions by JC-Fe_3_O_4_ and CT-Fe_3_O_4_ NPs.

There are reports in the existing literature that MNPs have the potential to adsorb heavy as well as toxic metal ions such as Hg^2+^,Cd^2+^,Pb^2+^,Co^2+^,Cu^2+^, etc. [43,58,59,60]. We have investigated the ability to remove toxic metal ions Co^2+^ and Cu^2+^ from aqueous medium via adsorption onto the synthesized JC-Fe_3_O_4_ and CT-Fe_3_O_4_ NPs. To understand the nature of adsorption of the metal ions, we applied our experimental data to the Langmuir and Freundlich adsorption isotherms; the results are presented in Figure 7a (for Co^2+^), Figure 7b (for Cu^2+^), and in Table 3. Table 4 represents a compilation of literature value of adsorption capacity of different adsorbent for Co^2+^ and Cu^2+^. From Table 3, it is evident that the values of the correlation coefficient (*R*^2^) for all the ions are greater for Langmuir plot than for Freundlich case, implying that the metal ion adsorption process follows the Langmuir model better than the Freundlich model. The observed maximum adsorption capacity (*Qm*) of CT-Fe_3_O_4_ NPs is 513.7 and 463.23 mg/g for Co^2+^ and Cu^2+^ respectively. The corresponding values are 501.3 and 543.3 mg/g for JC-Fe_3_O_4_. These values are much better than all other reported values, as seen in Table 4.

### 3.9. Magnetic Properties

**Figure 8:**
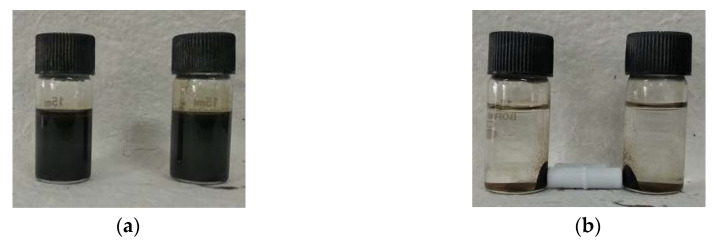

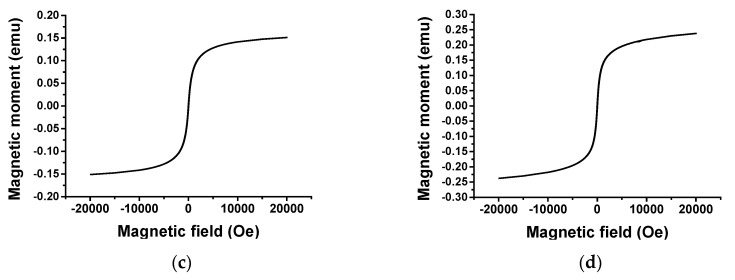
Respective MNPs solutions; (**a**) before separation; (**b**) after using a magnetic bar. VSM analysis of magnetite nanoparticles: (**c**) JC-Fe_3_O_4_ and (**d**) CT-Fe_3_O_4_ NPs.

In order to show the magnetic behavior of JC-Fe_3_O_4_ and CT-Fe_3_O_4_ NPs, the dispersed solutions (Figure 8a) were treated with magnet externally, and the nanoparticles were found to get deposited near the magnet (Figure 8b). This observation also exhibited the possibility of using the powerful magnetic field for the separation of MNPs after wastewater treatment.

Magnetic properties of the synthesized JC-Fe_3_O_4_ and CT-Fe_3_O_4_ NPs were also studied with the help of VSM (Vibrating sample magnetometer). Figure 8c,d gives the changes in the magnetization with the applied magnetic field. The superparamagnetic natures of the nanoparticles were confirmed by the absence of the hysteresis loop. The saturation magnetization for JC-Fe_3_O_4_ and CT-Fe_3_O_4_ NPs was found to be 38.46 and 34.35 emu/g, respectively.

### 3.10. Antibacterial Assay

#### 3.10.1. Characterization of Bacteria Isolated from Pond Water

The collected pond water (without nanoparticles) was serially diluted, a single colony was isolated, and pure culture was generated. Gram staining was performed on the isolated bacterial culture and was found to be Gram-positive bacteria.

#### 3.10.2. Wastewater Treatment

**Figure 9:**
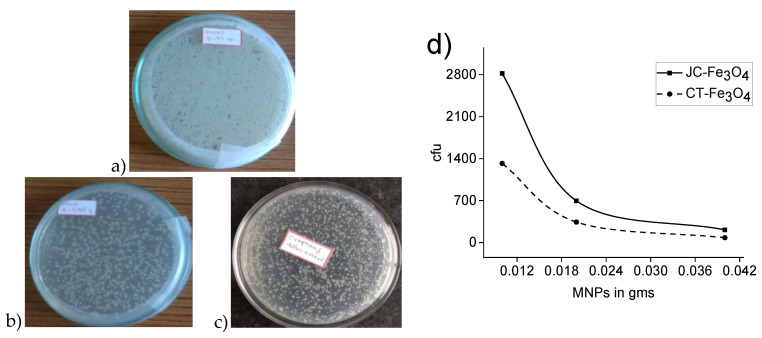
Images of bacterial culture: (**a**) untreated pond water; (**b**) with CT-Fe_3_O_4_; (**c**) with JC-Fe_3_O_4_; (**d**) bacterial concentration with respect to amount of MNPs.

Fe_3_O_4_ NPs had been previously reported in the literature to exhibit antibacterial activity [69,70,71]. Similarly, CT leaves were also reported to show antibacterial efficacy [35] as well as JC latex [53,72]. Hence, the antibacterial activity of CT-Fe_3_O_4_ and JC-Fe_3_O_4_ NPs were examined. The amount of bacteria colony was observed to be reduced by more than 50% in the case of the pond water treated with CT-Fe_3_O_4_ (Figure 9b) and JC-Fe_3_O_4_ NPs (Figure 9c) compared to the pond water that was not treated (Figure 9a) with any of the antibacterial agents. The CFU value also showed that the amount of bacteria colony of water treated with nanoparticles was being reduced with increasing the amount of nanoparticles. These observations confirmed the antibacterial activity of CT-Fe_3_O_4_ and JC-Fe_3_O_4_ against various types of water-borne bacteria (Figure 9d).

#### 3.10.3. Disk Diffusion

**Figure 10:**
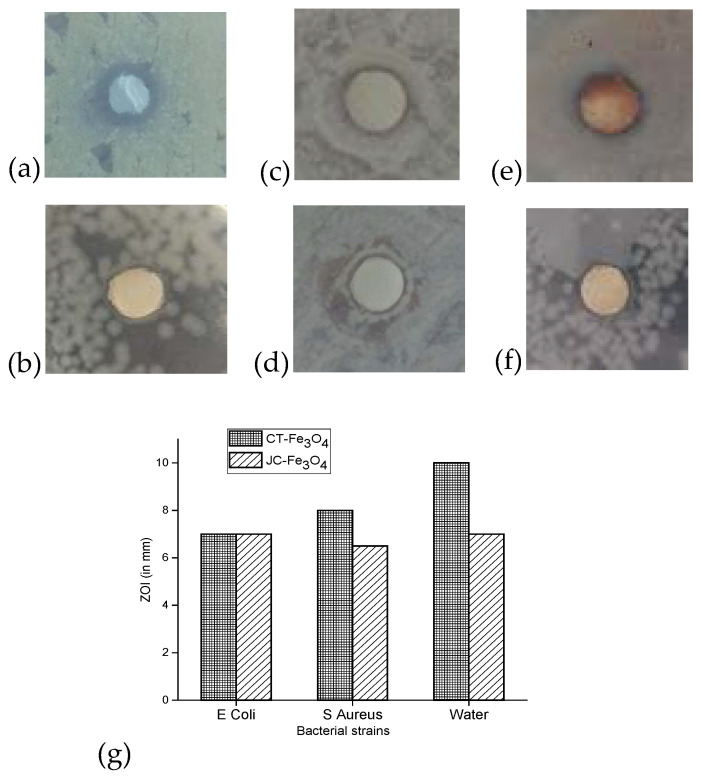
ZOI against water-born Gram-positive bacteria using (**a**) CT-Fe_3_O_4_ NPs (10 mm diameter) and (**b**) JC-Fe_3_O_4_ NPs (7 mm diameter); ZOI against *E. coli* (Gram-negative bacteria) (**c**) CT-Fe_3_O_4_ NPs (7 mm diameter)and (**d**) JC-Fe_3_O_4_ NPs (7 mm diameter); ZOI against *S. aureus* (Gram-positive bacteria) (**e**) CT-Fe_3_O_4_ NPs (8 mm diameter) and (**f**) JC-Fe_3_O_4_ NPs (6.5 mm diameter). (**g**) Relative zone of inhibition of CT-Fe_3_O_4_ and JC-Fe_3_O_4_ NPs against different bacteria.

The antibacterial activities of CT-Fe_3_O_4_ and JC-Fe_3_O_4_ NPs measured in terms of zone of inhibition (ZOI) are shown in Figure 10. It was observed that ZOI against water-born Gram-positive bacteria for CT-Fe_3_O_4_ and JC-Fe_3_O_4_ NPs showed a diameter of 10 mm and 7 mm respectively (Figure 10a,b). In the case of *E. coli*, ZOI was found to be the same (i.e., 7 mm) for both MNPs (Figure 10c,d). However, against *S. aureus* for CT-Fe_3_O_4_ the ZOI was 8 mm, whereas for CT-Fe_3_O_4_ it was 6.5 mm (Figure 10e,f). Based on the above result, it was observed that both CT-Fe_3_O_4_ and JC-Fe_3_O_4_ NPs exhibited quite effective antibacterial property against both Gram-positive and Gram-negative bacteria. The relative antibacterial activity of the two synthesized nanoparticles has been summarized in Figure 10g.

The result of the disk diffusion test showed that CT-Fe_3_O_4_ NPs exhibited better antibacterial activity than that of JC-Fe_3_O_4_ NPs. JC-Fe_3_O_4_ showed similar results for Gram-positive and Gram-negative bacteria, but CT-Fe_3_O_4_ was shown to be more effective against Gram-positive bacteria.

#### 3.10.4. MIC of CT-Fe_3_O_4_ and JC-Fe_3_O_4_NPs

**Figure 11:**
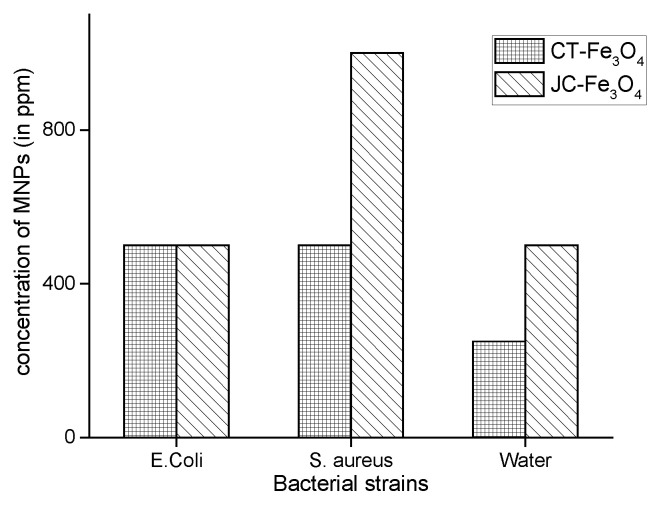
Minimum Inhibitory Concentration (MIC) for different bacteria.

MIC values of CT-Fe_3_O_4_ and JC-Fe_3_O_4_ against gram-positive and gram-negative bacteria are shown in Figure 11. For *E. coli* (gram-negative bacteria), the MIC value for both CT-Fe_3_O_4_ and JC-Fe_3_O_4_ was observed to be 500 ppm. Against *S. aureus* (gram-positive bacteria), the MIC value for CT-Fe_3_O_4_ and JC-Fe_3_O_4_ was 500 ppm and 1000 ppm respectively. In this regard, against the water-borne isolated Gram-positive bacteria, for CT-Fe_3_O_4_, the MIC was 250 ppm and for JC-Fe_3_O_4_ it was 500 ppm (Figure 11).

From the MIC, we got the expected results similar to disk diffusion test i.e., both CT-Fe_3_O_4_ and JC-Fe_3_O_4_ NPs, which showed decent antibacterial property. CT-Fe_3_O_4_ NPs were found to be more effective than JC-Fe_3_O_4_ NPs against both the Gram-positive and Gram-negative bacteria.

### 3.11. DPPH Scavenging Assay

**Figure 12:**
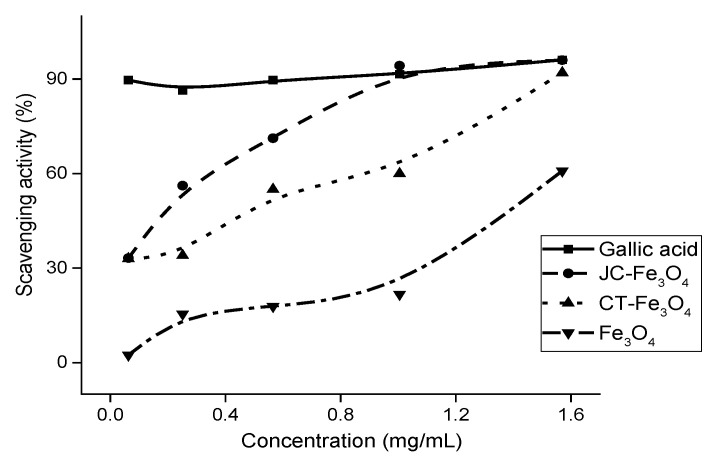
DPPH scavenging activity (%) of MNPs and standard gallic acid solution.

The antioxidant properties of both the nanoparticles (JC-Fe_3_O_4_ and CT-Fe_3_O_4_NPs) are shown in Figure 12. The DPPH scavenging assay of the respective MNPs resulted in IC_50_ values of 0.30 mg/mL for JC-Fe_3_O_4_, and for CT-Fe_3_O_4_ it was 0.67 mg/mL; and the IC_50_ value for the uncoated Fe_3_O_4_ nanoparticle was estimated to be 1.40 mg/mL. The chosen standard (positive control) was the gallic acid solution in methanol. All the concentrations were taken as 0.06 mg/mL, 0.25 mg/mL, 0.57 mg/mL, 1.00 mg/mL, and 1.57 mg/mL in methanol.

### 3.12. Measurement of Cytotoxicity Using MTT Assay

**Figure 13:**
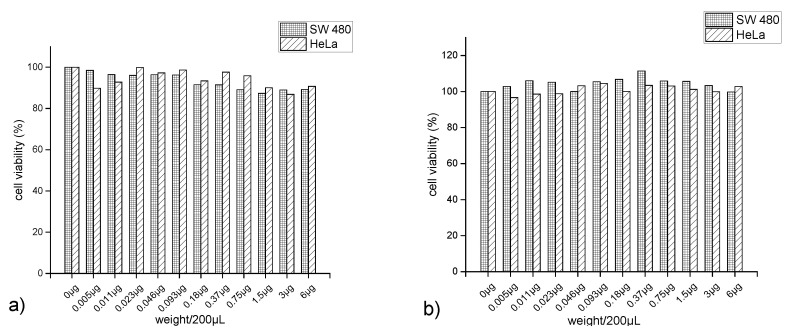
In vitro cytotoxicity of (**a**) JC-Fe_3_O_4_ NPs and (**b**) CT-Fe_3_O_4_ NPs against SW480 and HeLa cells assessed by MTT assays. Cytotoxicity is given as the percentage of viable cells remaining after treatment.

In addition to more efficient water purifying capabilities of the natural product-coated MNPs, it is important to study the overall toxicity associated with them. Since treated water, may consist of residual MNPs in ppm level due to inefficient removal process. It is reported that coated MNPs also showed lower cytotoxicity towards cancerous cells than the uncoated one [73]. Hence, to investigate whether the synthesized coated MNPs are toxic to human cells, cytotoxicity was investigated against human cancer cell lines (SW480 and HeLa) by MTT assay. Each cell line was incubated with both the MNPs for 48 h in different concentrations (Figure 13), and then the percentage viability of the cells was estimated. The percentage viability of the cells was found to be little enhanced in CT-Fe_3_O_4_ and almost remained the same in JC-Fe_3_O_4_. Based on the in vitro cytotoxicity results, it can be concluded that the MNPs did not exhibit cytotoxicity towards both cell lines, indicating these MNPs are not harmful to human cells. These results were well corroborated with the previous literature with natural product-coated MNPs [74].

## 4. Conclusions

The present study reports the green syntheses of two natural products, coated JC-Fe_3_O_4_ and CT-Fe_3_O_4_ NPs. Both the synthesized MNPs are effective in removing the content of wastewater like organic dyes and toxic metal ions. The study also shows that the nanoparticles are effective as antibacterial agents (both Gram-positive and Gram-negative bacteria) as well as antioxidant agents. Both the coated MNPs also do not exhibit any cytotoxic effect, as shown by the MTT assay. Therefore, JC-Fe_3_O_4_ and CT-Fe_3_O_4_ NPs both show promise for environment-friendly composites for effective water treatment.

## Figures and Tables

**Table 1:** Adsorption isotherm parameters for MB dye onto JC-Fe_3_O_4_ adsorbents.

Adsorbent	Adsorbate	Langmuir Isotherm				Freundlich Isotherm		
		*Qm*	*K_L_*	*R_L_*	*R^2^*	*K_F_*	1/*n*	*R^2^*
JC-Fe_3_O_4_	MB dye	466.6	0.0078	0.204-0.340	0.919	17.248	0.539	0.979

**Table 2:** Kinetics data with correlation coefficients for MB dye onto JC-Fe_3_O_4._

Adsorbent	Adsorbate	*Q_e_*(expt) (mg/g)	Pseudo-First Order			Pseudo-Second Order		
			*Q_e_*(cal) (mg/g)	*K*_1_ (min^−1^)	*R* ^2^	*Q_e_*(cal) (mg/g)	*K*_2_(g/mg·min)	*R* ^2^
**JC-Fe_3_O_4_**	**MB dye**	96.25	57.67	0.0368	0.980	104.82	8.02 × 10^−4^	0.998

**Table 3:** Adsorption isotherm parameters for metal ions onto JC-Fe_3_O_4_ and CT-Fe_3_O_4_ adsorbents.

Adsorbent	Adsorbate	Langmuir Isotherm				Freundlich Isotherm		
		*Q_m_*	*K_L_*	*R_L_*	*R* ^2^	*K_F_*	1/*n*	*R* ^2^
JC-Fe_3_O_4_	Cu^2+^	543.3	0.055	0.039–0.120	0.974	1.079	0.226	0.861
	Co^2+^	501.3	0.076	0.036–0.117	0.977	1.062	0.310	0.836
CT-Fe_3_O_4_	Cu^2+^	463.24	0.059	0.030–0.130	0.954	1.086	0.159	0.697
	Co^2+^	513.7	0.038	0.070–0.209	0.956	1.222	0.312	0.916

**Table 4:** Comparison of maximum adsorption capacity of CT-Fe_3_O_4_ and JC-Fe_3_O_4_ with other magnetite nanoparticle adsorbents literature values.

Adsorbate	Adsorbent	Adsorption Capacity (mg/g)	Ref.
Cu^2+^	Iron oxide nanoparticles	17.6	[61]
	GA-MNP	38.5	[61]
	Amino functionalized magnetic nanosorbent	12.4	[62]
	Fe_3_O_4_/AC	2.7	[63]
	CT-Fe_3_O_4_	463.2	Present study
	JC-Fe_3_O_4_	543.3	Present study
Co^2+^	Magnetite-citric acid nanoadsorbent	43.3	[64]
	MgFe_2_O_4_	135.5	[65]
	CT-Fe_3_O_4_	513.7	Present study
	JC-Fe_3_O_4_	501.3	Present study
MB dye	MNPs-POLP	128.2	[66]
	Magnetite/pectin NPs	125	[67]
	Magnetite/silica/pectin NPs	178.6	[67]
	Fe_3_O_4_ NPs coated with pectin and crosslinked with adipic acid (FN-PAA)	221.7	[68]
	JC-Fe_3_O_4_	466.6	Present study
